# 5-Amino-6-methyl­quinolin-1-ium hydrogen malonate–malonic acid (2/1)

**DOI:** 10.1107/S1600536813002547

**Published:** 2013-02-02

**Authors:** Kaliyaperumal Thanigaimani, Nuridayanti Che Khalib, Suhana Arshad, Ibrahim Abdul Razak

**Affiliations:** a School of Physics, Universiti Sains Malaysia, 11800 USM, Penang, Malaysia

## Abstract

The asymmetric unit of the title compound, 2C_10_H_11_N_2_^+^·2C_3_H_3_O_4_^−^·C_3_H_4_O_4_, consists of one 5-amino-6-methyl­quinolin-1-ium cation, one hydrogen malonate (2-carb­oxy­acetate) anion and one-half mol­ecule of malonic acid which lies on a twofold rotation axis. The quinoline ring system is essentially planar, with a maximum deviation of 0.062 (2) Å for all non-H atoms. In the anion, an intra­molecular O—H⋯O hydrogen bond generates an *S*(6) ring. In the crystal, the components are linked *via* N—H⋯O and O—H⋯O hydrogen bonds into layers parallel to the *ac* plane. The crystal structure also features weak C—H⋯O hydrogen bonds and a π–π stacking inter­action with a centroid–centroid distance of 3.8189 (10) Å.

## Related literature

For background to and the biological activity of quinoline derivatives, see: Sasaki *et al.* (1998 ▶); Reux *et al.* (2009 ▶); Morimoto *et al.* (1991 ▶); Markees *et al.* (1970 ▶). For related structures, see: Thanigaimani *et al.* (2013*a* ▶,*b* ▶); Loh *et al.* (2010 ▶). For hydrogen-bond motifs, see: Bernstein *et al.* (1995 ▶). For bond-length data, see: Allen *et al.* (1987 ▶). 
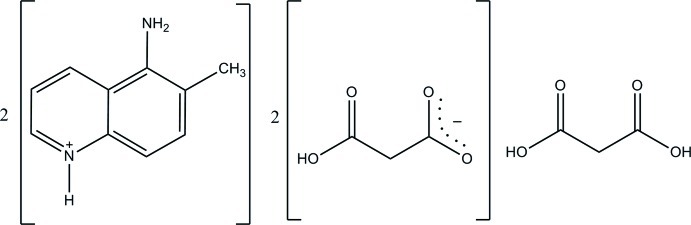


## Experimental

### 

#### Crystal data

2C_10_H_11_N_2_^+^·2C_3_H_3_O_4_^−^·C_3_H_4_O_4_
*M* *_r_* = 628.59
Monoclinic, 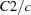

*a* = 24.701 (2) Å
*b* = 4.8530 (4) Å
*c* = 25.063 (2) Å
β = 95.321 (3)°
*V* = 2991.4 (4) Å^3^
*Z* = 4
Mo *K*α radiation
μ = 0.11 mm^−1^
*T* = 297 K
0.35 × 0.24 × 0.09 mm

#### Data collection

Bruker SMART APEXII DUO CCD area-detector diffractometer
Absorption correction: multi-scan (*SADABS*; Bruker, 2009 ▶) *T*_min_ = 0.962, *T*_max_ = 0.990
25870 measured reflections
3835 independent reflections
2644 reflections with *I* > 2σ(*I*)
*R*_int_ = 0.038

#### Refinement

*R*[*F* ^2^ > 2σ(*F* ^2^)] = 0.046
*wR*(*F* ^2^) = 0.130
*S* = 1.03
3835 reflections
225 parameters
1 restraint
H atoms treated by a mixture of independent and constrained refinement
Δρ_max_ = 0.18 e Å^−3^
Δρ_min_ = −0.20 e Å^−3^

### 

Data collection: *APEX2* (Bruker, 2009 ▶); cell refinement: *SAINT* (Bruker, 2009 ▶); data reduction: *SAINT*; program(s) used to solve structure: *SHELXTL* (Sheldrick, 2008 ▶); program(s) used to refine structure: *SHELXTL*; molecular graphics: *SHELXTL*; software used to prepare material for publication: *SHELXTL* and *PLATON* (Spek, 2009 ▶).

## Supplementary Material

Crystal structure: contains datablock(s) global, I. DOI: 10.1107/S1600536813002547/is5233sup1.cif

Structure factors: contains datablock(s) I. DOI: 10.1107/S1600536813002547/is5233Isup2.hkl

Supplementary material file. DOI: 10.1107/S1600536813002547/is5233Isup3.cml

Additional supplementary materials:  crystallographic information; 3D view; checkCIF report

## Figures and Tables

**Table 1:** Hydrogen-bond geometry (Å, °)

*D*—H⋯*A*	*D*—H	H⋯*A*	*D*⋯*A*	*D*—H⋯*A*
N1—H1*N*1⋯O3	1.00 (2)	1.76 (2)	2.7450 (17)	172.3 (18)
O2—H1*O*2⋯O4	0.86 (1)	1.64 (1)	2.4630 (18)	159 (2)
C1—H1*A*⋯O4	0.93	2.44	3.095 (2)	127
N2—H1*N*2⋯O1^i^	0.91 (2)	2.11 (2)	3.012 (2)	173.4 (18)
N2—H2*N*2⋯O5^ii^	0.90 (2)	2.20 (2)	3.076 (2)	166 (2)
O6—H106⋯O3^iii^	1.02 (3)	1.60 (3)	2.5927 (19)	164 (2)
C1—H1*A*⋯O2^iv^	0.93	2.28	3.106 (2)	148
C3—H3*A*⋯O1^i^	0.93	2.34	3.2648 (19)	174

Symmetry codes: (i) 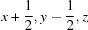; (ii) 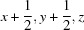; (iii) 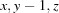; (iv) 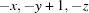.

**Fig. 1.**
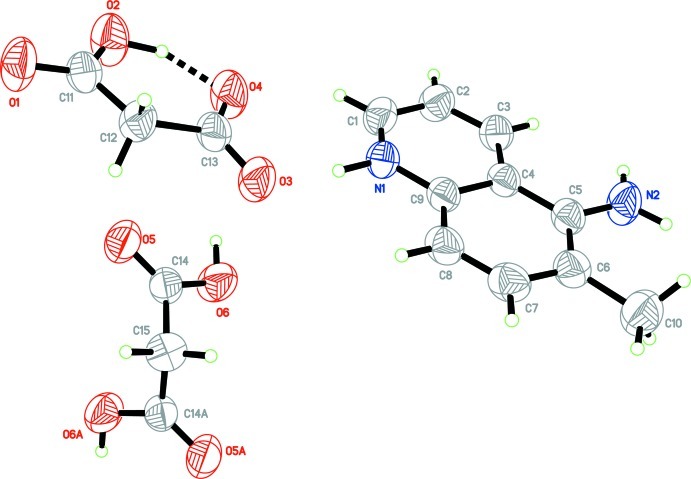
The molecular structure of the title compound with atom labels with 50% probability displacement ellipsoids.

**Fig. 2.**
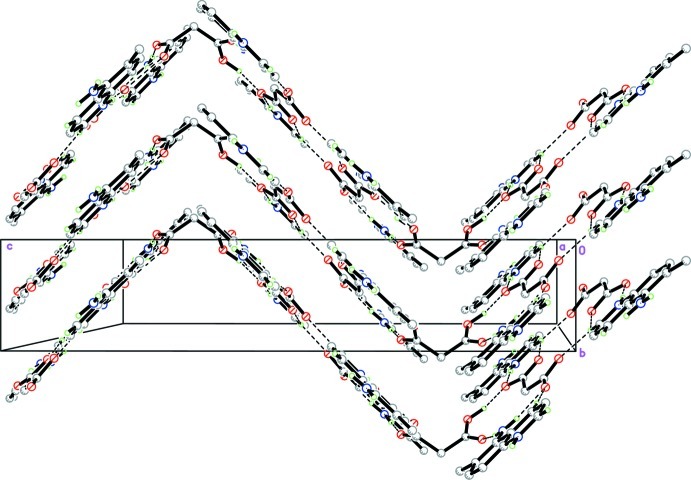
A crystal packing diagram of the title compound. H atoms not involved in the hydrogen bonds (dashed lines) have been omitted for clarity.

## supplementary crystallographic information

### Comment

Recently, hydrogen-bonding patterns involving quinoline and its derivatives with organic acid have been investigated (Thanigaimani *et al.*, 2013*a*,*b*; Loh *et al.*, 2010). Syntheses of the quinoline derivatives were discussed earlier (Sasaki *et al.*, 1998; Reux *et al.*, 2009). Quinolines and their derivatives are very important compounds because of their wide occurrence in natural products (Morimoto *et al.*, 1991) and biologically active compounds (Markees *et al.*, 1970). Herein we report the crystal structure and supramolecular patterns of the new compound containing quinoline derivative and malonic acid components.

The asymmetric unit of the title compound (Fig. 1) contains a protonated 5-amino-6-methylquinolin-1-ium cation, a hydrogen malonate anion and a half of the malonic acid molecule. The dihedral angles between the quinoline ring and the planes formed by the malonate and malonic acid molecule are 10.39 (5) and 27.08 (8)°, respectively. The planar malonic acid molecule is located on a two-fold rotation axis. In the malonic acid, the C14—O5 bond distance of 1.1973 (19) Å is much shorter than the C14—O6 bond distance of 1.312 (2) Å indicating that the carboxyl group is not deprotonated in the crystal structure. The 5-amino-6-methylquinolinium cation is essentially planar with a maximum deviation of 0.062 (2) Å for atom C10. In the cation, a wider than normal angle [C1—N1—C9 = 123.32 (13)°] is subtended at the protonated N1 atom. The bond lengths (Allen *et al.*, 1987) and angles are normal. The anion is stabilized by an intramolecular O2—H1O2···O4 hydrogen bond, which forms an S(6) ring motif (Bernstein *et al.*, 1995).

In the crystal packing (Fig. 2), the ion pairs and malonic acid molecules are linked *via* O6—H1O6···O3^iii^, N1—H1N1···O3, N2—H2N2···O5^ii^ and N2—H1N2···O1^i^ hydrogen bonds (symmetry codes in Table 1), forming a layer parallel to *ac* plane. The crystal structure is further stabilized by C1—H1A···O4, C1—H1A···O2^iv^ and C3—H3A···O1^i^ hydrogen bonds (symmetry codes in Table 1) and a π–π stacking interaction between the pyridine rings (N1/C1–C4/C9) and the benzene ring (C4–C9) (*x*, -1 + *y*, *z* and *x*, 1 + *y*, *z*) with a centroid–centroid distance of 3.8189 (10) Å

### Experimental

Hot methanol solutions (20 ml) of 5-amino-6-methylquinoline (39 mg, Aldrich) and malonic acid (26 mg, Aldrich) were mixed and warmed over a heating magnetic stirrer hotplate for a few minutes. The resulting solution was allowed to cool slowly at room temperature and crystals of the title compound (I) appeared after a few days.

### Refinement

O- and N-bound H atoms were located in a difference Fourier map. Atoms H1O6, H1N1, H1N2 and H2N2 were refined freely, while atom H1O2 was refined with a bond restraint O—H = 0.82 (1) Å [refined distance: O6—H1O6 = 1.02 (3) Å, O2—H1O2 = 0.859 (10) Å, N1—H1N1 = 0.99 (2) Å, N2—H1N2 = 0.91 (2) Å and N2—H2N2 = 0.90 (2) Å]. The remaining hydrogen atoms were positioned geometrically (C—H = 0.93–0.97 Å) and were refined using a riding model, with *U*_iso_(H) = 1.2 *U*_eq_(C) or 1.5*U*_eq_(methyl C). A rotating-group model was used for the methyl group. One outlier (-6 0 10) was omitted in the final refinement.

### Crystal data

**Table:**

2C_10_H_11_N_2_^+^·2C_3_H_3_O_4_^−^·C_3_H_4_O_4_	*F*(000) = 1320
*M**_r_* = 628.59	*D*_x_ = 1.396 Mg m^−^^3^
Monoclinic, *C*2/*c*	Mo *K*α radiation, λ = 0.71073 Å
Hall symbol: -C 2yc	Cell parameters from 5351 reflections
*a* = 24.701 (2) Å	θ = 3.3–25.6°
*b* = 4.8530 (4) Å	µ = 0.11 mm^−^^1^
*c* = 25.063 (2) Å	*T* = 297 K
β = 95.321 (3)°	Block, orange
*V* = 2991.4 (4) Å^3^	0.35 × 0.24 × 0.09 mm
*Z* = 4	

### Data collection

**Table:**

Bruker SMART APEXII DUO CCD area-detector diffractometer	3835 independent reflections
Radiation source: fine-focus sealed tube	2644 reflections with *I* > 2σ(*I*)
Graphite monochromator	*R*_int_ = 0.038
φ and ω scans	θ_max_ = 28.6°, θ_min_ = 1.6°
Absorption correction: multi-scan (*SADABS*; Bruker, 2009)	*h* = −31→32
*T*_min_ = 0.962, *T*_max_ = 0.990	*k* = −6→6
25870 measured reflections	*l* = −33→33

### Refinement

**Table:**

Refinement on *F*^2^	Primary atom site location: structure-invariant direct methods
Least-squares matrix: full	Secondary atom site location: difference Fourier map
*R*[*F*^2^ > 2σ(*F*^2^)] = 0.046	Hydrogen site location: inferred from neighbouring sites
*wR*(*F*^2^) = 0.130	H atoms treated by a mixture of independent and constrained refinement
*S* = 1.03	*w* = 1/[σ^2^(*F*_o_^2^) + (0.0604*P*)^2^ + 1.0717*P*] where *P* = (*F*_o_^2^ + 2*F*_c_^2^)/3
3835 reflections	(Δ/σ)_max_ < 0.001
225 parameters	Δρ_max_ = 0.18 e Å^−^^3^
1 restraint	Δρ_min_ = −0.20 e Å^−^^3^

### Special details

**Table:**

Geometry. All e.s.d.'s (except the e.s.d. in the dihedral angle between two l.s. planes) are estimated using the full covariance matrix. The cell e.s.d.'s are taken into account individually in the estimation of e.s.d.'s in distances, angles and torsion angles; correlations between e.s.d.'s in cell parameters are only used when they are defined by crystal symmetry. An approximate (isotropic) treatment of cell e.s.d.'s is used for estimating e.s.d.'s involving l.s. planes.
Refinement. Refinement of *F*^2^ against ALL reflections. The weighted *R*-factor *wR* and goodness of fit *S* are based on *F*^2^, conventional *R*-factors *R* are based on *F*, with *F* set to zero for negative *F*^2^. The threshold expression of *F*^2^ > σ(*F*^2^) is used only for calculating *R*-factors(gt) *etc*. and is not relevant to the choice of reflections for refinement. *R*-factors based on *F*^2^ are statistically about twice as large as those based on *F*, and *R*- factors based on ALL data will be even larger.

### Fractional atomic coordinates and isotropic or equivalent isotropic displacement parameters (Å^2^)

**Table:**

	*x*	*y*	*z*	*U*_iso_*/*U*_eq_	
O1	−0.13586 (5)	0.9772 (3)	0.04300 (6)	0.0754 (4)	
O2	−0.07247 (5)	0.7142 (3)	0.01501 (6)	0.0746 (4)	
O3	0.04562 (4)	1.0469 (3)	0.11941 (5)	0.0646 (3)	
O4	0.02278 (5)	0.7599 (3)	0.05274 (5)	0.0690 (4)	
O5	−0.04221 (5)	0.5560 (3)	0.16694 (5)	0.0732 (4)	
O6	0.03881 (5)	0.3951 (3)	0.19696 (5)	0.0709 (4)	
N1	0.14556 (5)	0.8482 (3)	0.09860 (5)	0.0512 (3)	
N2	0.34061 (6)	0.9024 (4)	0.12530 (7)	0.0632 (4)	
C1	0.14687 (6)	0.6550 (4)	0.06193 (7)	0.0565 (4)	
H1A	0.1144	0.5844	0.0456	0.068*	
C2	0.19582 (6)	0.5552 (4)	0.04724 (7)	0.0565 (4)	
H2A	0.1966	0.4172	0.0216	0.068*	
C3	0.24305 (6)	0.6638 (3)	0.07126 (6)	0.0513 (4)	
H3A	0.2762	0.5997	0.0614	0.062*	
C4	0.24260 (5)	0.8699 (3)	0.11047 (6)	0.0444 (3)	
C5	0.29079 (6)	0.9876 (3)	0.13779 (6)	0.0475 (3)	
C6	0.28603 (6)	1.1839 (4)	0.17747 (6)	0.0535 (4)	
C7	0.23408 (7)	1.2695 (4)	0.18874 (7)	0.0577 (4)	
H7A	0.2314	1.4035	0.2149	0.069*	
C8	0.18716 (6)	1.1662 (4)	0.16329 (6)	0.0546 (4)	
H8A	0.1534	1.2292	0.1716	0.066*	
C9	0.19147 (6)	0.9639 (3)	0.12455 (6)	0.0456 (3)	
C10	0.33584 (8)	1.3009 (5)	0.20828 (8)	0.0752 (6)	
H10A	0.3588	1.3832	0.1838	0.113*	
H10B	0.3553	1.1561	0.2278	0.113*	
H10C	0.3253	1.4381	0.2329	0.113*	
C11	−0.08862 (6)	0.9118 (3)	0.04500 (6)	0.0519 (4)	
C12	−0.04600 (6)	1.0564 (4)	0.08107 (7)	0.0542 (4)	
H12A	−0.0578	1.0564	0.1169	0.065*	
H12B	−0.0449	1.2470	0.0696	0.065*	
C13	0.01154 (6)	0.9451 (3)	0.08464 (7)	0.0518 (4)	
C14	−0.00419 (6)	0.5507 (3)	0.20023 (6)	0.0508 (4)	
C15	0.0000	0.7256 (5)	0.2500	0.0577 (6)	
H15A	−0.0318	0.8431	0.2497	0.069*	
H1N2	0.3459 (8)	0.782 (5)	0.0986 (8)	0.076 (6)*	
H1N1	0.1100 (8)	0.914 (4)	0.1093 (8)	0.076 (6)*	
H2N2	0.3720 (10)	0.970 (5)	0.1407 (9)	0.087 (7)*	
H1O2	−0.0377 (4)	0.708 (5)	0.0215 (9)	0.101 (8)*	
H106	0.0359 (11)	0.277 (6)	0.1633 (11)	0.118 (9)*	

### Atomic displacement parameters (Å^2^)

**Table:**

	*U*^11^	*U*^22^	*U*^33^	*U*^12^	*U*^13^	*U*^23^
O1	0.0428 (6)	0.0887 (9)	0.0941 (9)	0.0145 (6)	0.0024 (6)	−0.0245 (8)
O2	0.0540 (7)	0.0738 (8)	0.0968 (10)	0.0054 (6)	0.0106 (7)	−0.0355 (7)
O3	0.0419 (6)	0.0825 (8)	0.0691 (7)	0.0156 (6)	0.0030 (5)	−0.0146 (6)
O4	0.0498 (6)	0.0737 (8)	0.0849 (8)	0.0168 (6)	0.0147 (6)	−0.0213 (7)
O5	0.0489 (6)	0.0961 (10)	0.0726 (8)	0.0098 (6)	−0.0045 (6)	−0.0194 (7)
O6	0.0606 (7)	0.0848 (9)	0.0654 (8)	0.0233 (7)	−0.0046 (6)	−0.0097 (7)
N1	0.0367 (6)	0.0633 (8)	0.0538 (7)	0.0116 (6)	0.0049 (5)	0.0025 (6)
N2	0.0379 (7)	0.0767 (10)	0.0755 (10)	0.0060 (7)	0.0072 (7)	−0.0156 (8)
C1	0.0425 (8)	0.0668 (10)	0.0592 (9)	0.0066 (7)	−0.0009 (7)	0.0000 (8)
C2	0.0486 (8)	0.0628 (10)	0.0578 (9)	0.0086 (7)	0.0039 (7)	−0.0074 (8)
C3	0.0419 (8)	0.0590 (9)	0.0537 (9)	0.0118 (7)	0.0084 (6)	−0.0010 (7)
C4	0.0382 (7)	0.0500 (8)	0.0457 (7)	0.0089 (6)	0.0080 (6)	0.0074 (6)
C5	0.0400 (7)	0.0524 (8)	0.0507 (8)	0.0071 (6)	0.0076 (6)	0.0063 (7)
C6	0.0490 (8)	0.0601 (9)	0.0522 (9)	0.0032 (7)	0.0084 (6)	0.0002 (7)
C7	0.0597 (10)	0.0624 (10)	0.0525 (9)	0.0095 (8)	0.0131 (7)	−0.0059 (8)
C8	0.0469 (8)	0.0654 (10)	0.0530 (9)	0.0148 (7)	0.0127 (7)	0.0013 (7)
C9	0.0380 (7)	0.0535 (8)	0.0457 (8)	0.0103 (6)	0.0068 (6)	0.0090 (6)
C10	0.0613 (11)	0.0880 (14)	0.0763 (12)	−0.0041 (10)	0.0059 (9)	−0.0216 (11)
C11	0.0458 (8)	0.0521 (8)	0.0588 (9)	0.0081 (7)	0.0097 (7)	−0.0027 (7)
C12	0.0461 (8)	0.0580 (9)	0.0580 (9)	0.0166 (7)	0.0023 (7)	−0.0075 (7)
C13	0.0422 (8)	0.0588 (9)	0.0555 (9)	0.0132 (7)	0.0104 (7)	0.0015 (7)
C14	0.0442 (8)	0.0558 (9)	0.0529 (8)	0.0000 (7)	0.0066 (6)	0.0047 (7)
C15	0.0634 (14)	0.0508 (12)	0.0581 (13)	0.000	0.0020 (11)	0.000

### Geometric parameters (Å, º)

**Table:**

O1—C11	1.2060 (18)	C4—C9	1.4178 (19)
O2—C11	1.3037 (19)	C4—C5	1.436 (2)
O2—H1O2	0.859 (10)	C5—C6	1.390 (2)
O3—C13	1.2556 (19)	C6—C7	1.402 (2)
O4—C13	1.2511 (19)	C6—C10	1.502 (2)
O5—C14	1.1973 (19)	C7—C8	1.365 (2)
O6—C14	1.3119 (19)	C7—H7A	0.9300
O6—H106	1.02 (3)	C8—C9	1.392 (2)
N1—C1	1.316 (2)	C8—H8A	0.9300
N1—C9	1.374 (2)	C10—H10A	0.9600
N1—H1N1	0.99 (2)	C10—H10B	0.9600
N2—C5	1.3619 (19)	C10—H10C	0.9600
N2—H1N2	0.91 (2)	C11—C12	1.497 (2)
N2—H2N2	0.90 (2)	C12—C13	1.515 (2)
C1—C2	1.384 (2)	C12—H12A	0.9700
C1—H1A	0.9300	C12—H12B	0.9700
C2—C3	1.368 (2)	C14—C15	1.504 (2)
C2—H2A	0.9300	C15—C14^i^	1.505 (2)
C3—C4	1.403 (2)	C15—H15A	0.9700
C3—H3A	0.9300		
			
C11—O2—H1O2	105.8 (17)	C7—C8—H8A	121.0
C14—O6—H106	112.4 (15)	C9—C8—H8A	121.0
C1—N1—C9	123.32 (13)	N1—C9—C8	120.35 (13)
C1—N1—H1N1	119.8 (12)	N1—C9—C4	117.77 (14)
C9—N1—H1N1	116.9 (12)	C8—C9—C4	121.88 (14)
C5—N2—H1N2	124.0 (13)	C6—C10—H10A	109.5
C5—N2—H2N2	123.7 (15)	C6—C10—H10B	109.5
H1N2—N2—H2N2	112.1 (19)	H10A—C10—H10B	109.5
N1—C1—C2	120.93 (15)	C6—C10—H10C	109.5
N1—C1—H1A	119.5	H10A—C10—H10C	109.5
C2—C1—H1A	119.5	H10B—C10—H10C	109.5
C3—C2—C1	118.60 (16)	O1—C11—O2	121.09 (16)
C3—C2—H2A	120.7	O1—C11—C12	121.68 (14)
C1—C2—H2A	120.7	O2—C11—C12	117.22 (13)
C2—C3—C4	121.41 (14)	C11—C12—C13	118.13 (14)
C2—C3—H3A	119.3	C11—C12—H12A	107.8
C4—C3—H3A	119.3	C13—C12—H12A	107.8
C3—C4—C9	117.96 (13)	C11—C12—H12B	107.8
C3—C4—C5	123.90 (13)	C13—C12—H12B	107.8
C9—C4—C5	118.13 (14)	H12A—C12—H12B	107.1
N2—C5—C6	120.70 (15)	O4—C13—O3	123.50 (14)
N2—C5—C4	119.77 (15)	O4—C13—C12	118.75 (15)
C6—C5—C4	119.52 (13)	O3—C13—C12	117.74 (14)
C5—C6—C7	119.12 (15)	O5—C14—O6	123.80 (16)
C5—C6—C10	120.49 (15)	O5—C14—C15	123.69 (14)
C7—C6—C10	120.39 (16)	O6—C14—C15	112.50 (13)
C8—C7—C6	123.39 (16)	C14—C15—C14^i^	111.30 (19)
C8—C7—H7A	118.3	C14—C15—H15A	109.4
C6—C7—H7A	118.3	C14^i^—C15—H15A	109.4
C7—C8—C9	117.92 (14)		
			
C9—N1—C1—C2	0.1 (3)	C6—C7—C8—C9	0.6 (3)
N1—C1—C2—C3	−0.7 (3)	C1—N1—C9—C8	−179.50 (15)
C1—C2—C3—C4	0.7 (3)	C1—N1—C9—C4	0.5 (2)
C2—C3—C4—C9	−0.2 (2)	C7—C8—C9—N1	178.46 (15)
C2—C3—C4—C5	178.70 (15)	C7—C8—C9—C4	−1.5 (2)
C3—C4—C5—N2	1.1 (2)	C3—C4—C9—N1	−0.4 (2)
C9—C4—C5—N2	−179.97 (14)	C5—C4—C9—N1	−179.35 (13)
C3—C4—C5—C6	−177.67 (14)	C3—C4—C9—C8	179.55 (14)
C9—C4—C5—C6	1.2 (2)	C5—C4—C9—C8	0.6 (2)
N2—C5—C6—C7	179.13 (16)	O1—C11—C12—C13	174.80 (16)
C4—C5—C6—C7	−2.1 (2)	O2—C11—C12—C13	−6.3 (2)
N2—C5—C6—C10	−1.5 (3)	C11—C12—C13—O4	8.8 (2)
C4—C5—C6—C10	177.31 (16)	C11—C12—C13—O3	−171.96 (15)
C5—C6—C7—C8	1.2 (3)	O5—C14—C15—C14^i^	119.16 (18)
C10—C6—C7—C8	−178.19 (18)	O6—C14—C15—C14^i^	−61.88 (12)

Symmetry code: (i) −*x*, *y*, −*z*+1/2.

### Hydrogen-bond geometry (Å, º)

**Table:**

*D*—H···*A*	*D*—H	H···*A*	*D*···*A*	*D*—H···*A*
N1—H1*N*1···O3	1.00 (2)	1.76 (2)	2.7450 (17)	172.3 (18)
O2—H1*O*2···O4	0.86 (1)	1.64 (1)	2.4630 (18)	159 (2)
C1—H1*A*···O4	0.93	2.44	3.095 (2)	127
N2—H1*N*2···O1^ii^	0.91 (2)	2.11 (2)	3.012 (2)	173.4 (18)
N2—H2*N*2···O5^iii^	0.90 (2)	2.20 (2)	3.076 (2)	166 (2)
O6—H106···O3^iv^	1.02 (3)	1.60 (3)	2.5927 (19)	164 (2)
C1—H1*A*···O2^v^	0.93	2.28	3.106 (2)	148
C3—H3*A*···O1^ii^	0.93	2.34	3.2648 (19)	174

Symmetry codes: (ii) *x*+1/2, *y*−1/2, *z*; (iii) *x*+1/2, *y*+1/2, *z*; (iv) *x*, *y*−1, *z*; (v) −*x*, −*y*+1, −*z*.
